# Management of undescended testis may be improved with educational updates and new transferring model

**DOI:** 10.1186/s13052-018-0499-4

**Published:** 2018-05-24

**Authors:** Wei Yi, Wu Sheng-de, Shen Lian-Ju, Lin Tao, He Da-wei, Wei Guang-hui

**Affiliations:** 1 Department of Urology, Children’s Hospital of Chongqing Medical University, Room 806, Kejiao Building (NO.6 Building), No.136, 2nd Zhongshan Road, Yuzhong District, Chongqing City, 400014 China; 2 Chongqing Key Laboratory of Child Urogenital Development and Tissue Engineering, Chongqing, China; 3 Ministry of Education Key Laboratory of Child Development and Disorders, Chongqing, China; 4 China International Science and Technology Cooperation base of Child development and Critical Disorders, Chongqing, China; 5 Chongqing Key Laboratory of Pediatrics Chongqing, Chongqing, China

**Keywords:** Children, Orchidopexy, Undescended testis (UDT), Age, Educational updates

## Abstract

**Background:**

To investigate whether management of undescended testis (UDT) may be improved with educational updates and new transferring model among referring providers (RPs).

**Methods:**

The age of orchidopexies performed in Children’s Hospital of Chongqing Medical University were reviewed. We then proposed educational updates and new transferring model among RPs. The age of orchidopexies performed after our intervention were collected. Data were represented graphically and statistical analysis Chi-square for trend were used.

**Results:**

A total of 1543 orchidopexies were performed. The median age of orchidopexy did not matched the target age of 6–12 months in any subsequent year. Survey of the RPs showed that 48.85% of their recommended age was below 12 months. However, only 25.50% of them would directly make a surgical referral to pediatric surgery specifically at this point. After we proposed educational updates, tracking the age of orchidopexy revealed a statistically significant trend downward.

**Conclusions:**

The management of undescended testis may be improved with educational updates and new transferring model among primary healthcare practitioners.

## Background

Undescended testis (UDT) is a common condition in childhood, and is related to endocrine disorders, sperm damage, and testicular deterioration [1, 2]. It is estimated to affect 1 to 4% of full term and up to 30% of preterm male neonates [3, 4]. The recommended age of orchiopexy changed to 6–12 months of age over a decade ago [5–8]. However, studies have also shown that the average age at orchidopexy currently remains significantly older than the suggested targets, and bringing the age down may be difficult to achieve [9, 10].

This problem is similar in the southwest district of China based on our clinical observation, and we believed that some aspects of management could be improved with educational updates for the referring provider (RPs)/ primary healthcare practitioners. A study at our surgery centers (one of the biggest children’s hospital in the southwest district of China) from April 2010 to July 2016 was undertaken. This study aimed to analyse the problem, proposing educational updates/new transferring model and determining if we could bring the age down.

## Methods

### Review of age at orchidopexy

Ethical approval was not required as this research was conducted on previously collected non-identifiable information.

### Part 1: Assessment of management of UDT prior to referral to urology

The age undergoing orchidopexy from 1 April 2010 to 1 February 2015 for undescended testes were included. Age at orchidopexy was calculated from date of birth and date of operation.

### Part 2: Assessment of the delay to orchidopexy

An anonymous questionnaire entitled “Cognition of cryptorchidism and referral patterns for children with undescended testes” was distributed to 373 RPs/ healthcare practitioners with a 100% response rate. The multiple choice questionnaire asked: how and at what age children with UDT were usually detected, the perceived optimal age for orchidopexy. Data were then collated and represented graphically.

The date diagnosed of UDT, the date referral to specialist center for outpatient appointment, the date of orchidopexy were collected from electronic appointment system.

We list the reasons why there may be a delay to orchidopexy for any given patients, then proposed educational updates and new transferring model.

### Part 3: Assessment of the effectiveness of educational services to RPs

All aspects of UDT management were addressed in our educational updates, and we chose to track the age of orchidopexy to determine if management could be improved. The following updates were provided: (1) A continuing medical education course on UDT management in January 2015 including lecture and training test in order to make them know about the issue, follow our suggested referral pathway for UDT. We proposed guideline for referral and management of undescended testes for early ochidopexy as follows: Healthcare practitioners help making referral of the given patients to specialist center for outpatient appointment before age 3 months if accurate diagnosis is performed. The cases diagnosed with undescended testis (not retractile testes) could undergo orchidopexy before age of 12 months; (2) Inserting a note that the recommended age of orchidopexy is 6–12 months, in August 2015; (3) An educational lecture named “referral as early as 3 months old” was held in February 2016. All providers who had referred a patient to our specialist center from April 2014 to January 2015 (Included the primary healthcare practitioners, and the providers from the other department of outpatient are educated.

The age undergoing orchidopexy for an UDT were then collected from 1 Feb 2015 to 30 July 2016 and 1 August 2016 to 1 August 2017 after our educational updates and new transferring model.

Data were represented graphically and statistic analysis Chi-square for trend were the statistic methods used to evaluate the change in age of orchidopexy over the study period.

## Results

### Part 1: Assessment of management of UDT prior to referral to urology

#### The age of orchidopexy

**Fig. 1:**
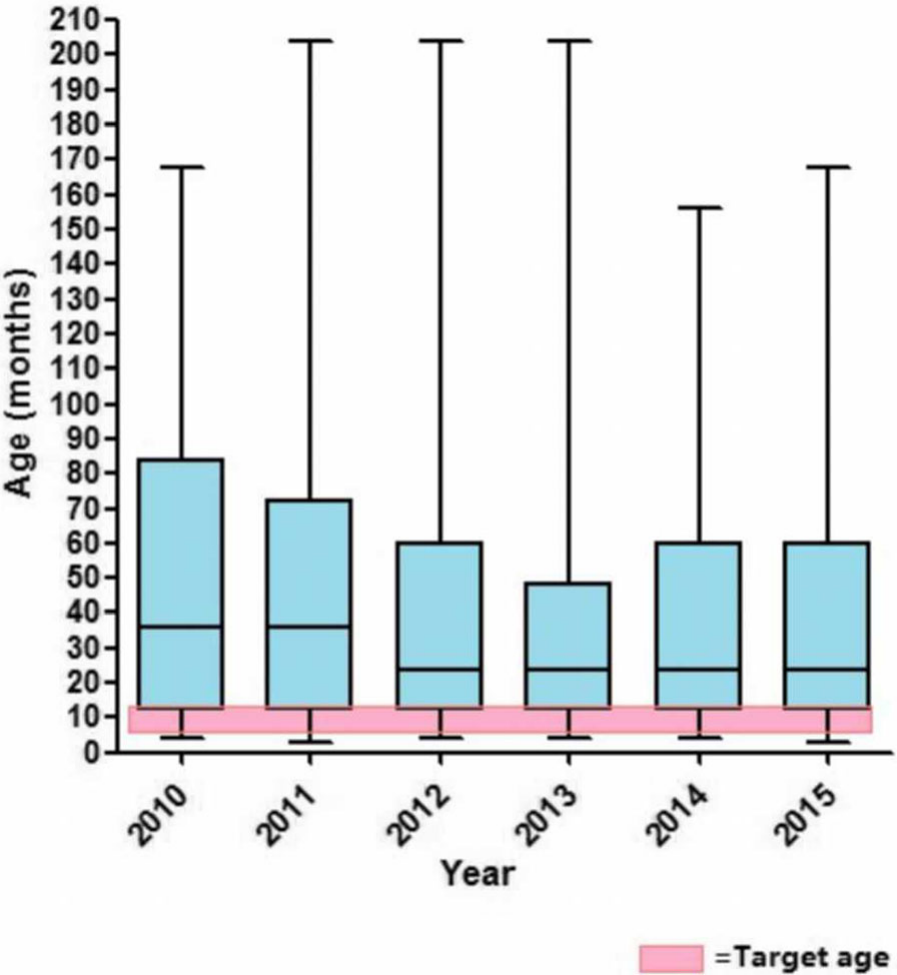
Graph showing the median age at orchidopexy each year from 2010 to 2015. Shaded box shows the target range for the age at orchidopexy during that time period

A total of 1543 orchidopexies were performed from 1 April 2010 to 1 February 2015. The median age of orchidopexy fell from 3 years in 2010 to 2 years in 2015. However, the median age has not matched the target age of 6–9 months in any subsequent year within the 5-year study period (Fig. 1).

### Part 2: Assessment of the delay to orchidopexy

#### 2.1 Survey of primary healthcare practitioners

The baby check is across the China, and within the primary healthcare practitioner practices surveyed, these checks of UDT were always performed by the healthcare practitioner themselves (100%) (Total number = 305). However, only 63.61% (194/305) of the primary healthcare practitioners would perform normal pediatric urology examination. We also noticed that only 41.75% (81/194) of them had correct pediatric urology examination. 15.41% (47/305) of the healthcare practitioners know the recommended age of orchidopexy is 6–9 months, 29.2%(89/305) is 9–12 months, 43.61% (133/305) is 12–24 months, 5.6%(17/305) of their referral time is 24–36 months and even 1.97%(6/305) of the healthcare practitioners lived in poor towns have no accurate ideas about the age of ochidopexy. 48.85% (149/305) of their recommended age is below 12 months. However, only 25.50% (38/149) of them would directly make a surgical referral to pediatric surgery specifically at this point. The majority of the primary healthcare practitioners would suggest bringing children back for reassessment, usually at around 1 year, and receive the operation before 2 years old if required.

#### 2.2 Study of waiting time

**Fig. 2:**
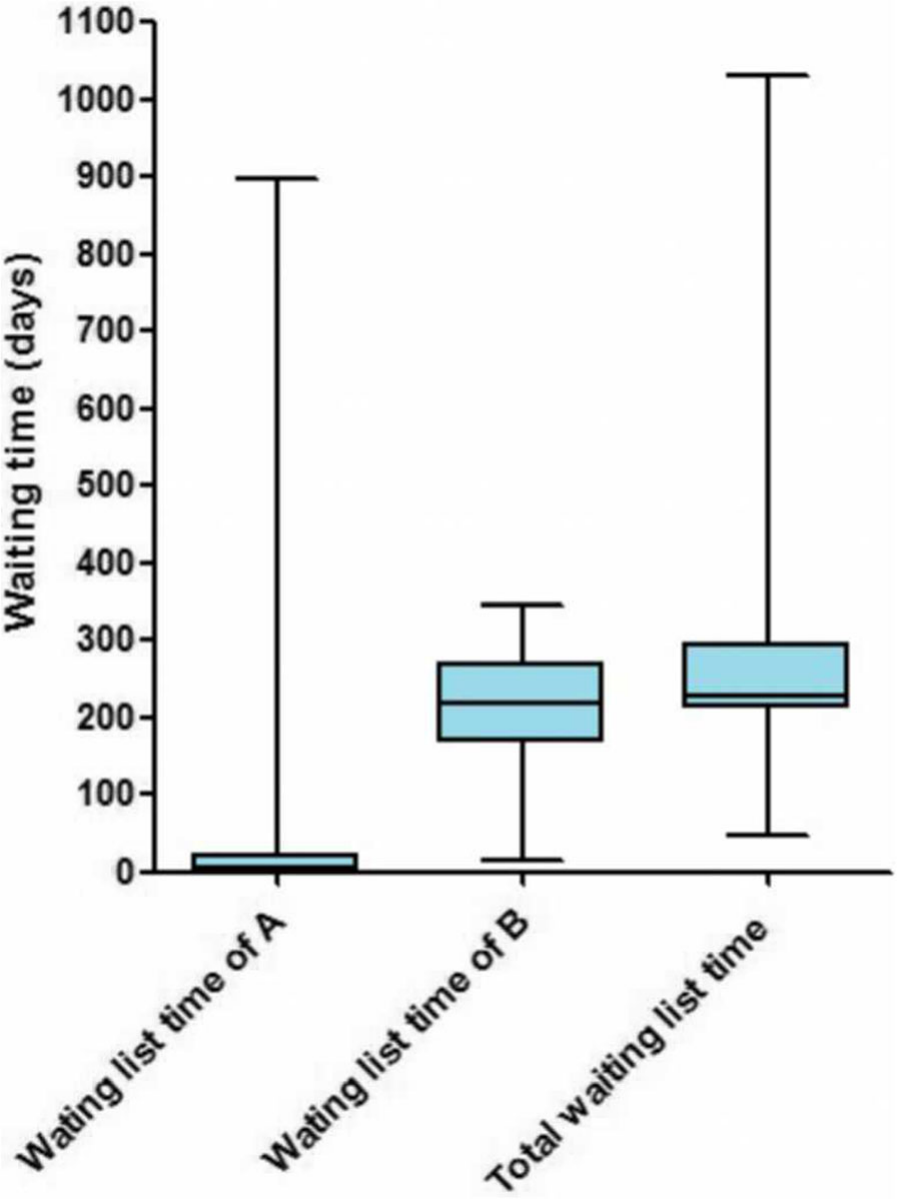
Graph showing the waiting list time of orchidopexies (*n* = 199). A = The time from diagnosing UDT to referral to specialist center. B = The time from making outpatient appointment to orchidopexy

The date diagnosed of UDT, the date referral to specialist center for outpatient appointment and the date of orchidopexy were collected from electronic appointment system. 199 of the total 1543 orchidopexies were included because of some missing information. The time from diagnosing UDT to referral to specialist center will waste 0 day to 897 days, and the time from making outpatient appointment to orchidopexy ranged from 75 days to 281 days. Overall, the total waiting list time of orchidopexy ranged from 79 days to 1032 days. The study has suggested that our pediatric surgery center need long waiting list times of about 7 months (the median time of total waiting list time is 226 days) (Fig. 2). Early orchidopexy would therefore require a referral before 3 months of age.

### Part 3: Assessment of the effectiveness of educational services to RPs

#### The age of orchidopexy

In the first stage, a total of 447 orchidopexies were performed in our hospital from 1 February 2015–30 July 2016. The median age at orchidopexy throughout this time period was analysed every 2 months, and it decreased from 30 months (Feb-Mar 2015, *n* = 56), 24 months (April–May 2015, *n* = 55), 24 months (July-Aug 2015, *n* = 55), 24 months (Aug-Sept 2015, *n* = 47), 30 months (Oct-Nov 2015, *n* = 49), 23 months (Dec 2015-Jan 2016, *n* = 49), 21 months (Feb-Mar 2016, *n* = 45), 13 months (April–May 2016, *n* = 53) to 12 months (June–July 2016, *n* = 38). Tracking the age of orchidopexy revealed a statistically significant trend downward after our educational update of new transferring model (*P*<0.05).

In the second stage, another 339 orchidopexies were performed in our hospital from 1 August 2016–1 August 2017. Median age at primary surgery decreased from 24 months in 2016 (February 2015–July 2016) to 21 months in 2017 (August 2016–August 2017).

Over all, 9.41%(74/786) had surgery by 1 year of age from February 2015 to 1 August 2017, yet the proportion increased slightly from 7.16% (32/447) in 2016 (February 2015–July 2016) to 12.39%(42/339) in 2017 (August 2016–August 2017).

However, only 33.92% (115/339) had surgery by 2 year of age in the second stage study when compared with 47.3%(211/447) in the first stage.

The decrease in percentage of orchidopexies performed before the age of 24 months was primarily owing to a considerable decrease in boys having surgery between 1 and 2 years of age, the proportion of which decreased from 40.04% (73/339) in 2016 (February 2015–July 2016) to 21.53%(179/447) in 2017 (August 2016–August 2017).

## Discussion

Undescended testis (UDT) is a common condition of childhood. Epidemiological data suggest that the incidence has not changed over the last 50 years. The main reasons for treatment of cryptorchidism include increased risks of impairment of fertility potential, testicular malignancy, torsion and/or associated inguinal hernia. Gonocyte maturation requires both normal testicular hormones and a temperature of around 33 °C. The disruption of gonocyte maturation results in the lower sperm counts seen in adult men who had UDT. In addition, the failure of gonocyte apoptosis may be responsible for the higher rates of malignancy seen with UDT [11]. The current standard of therapy is orchidopexy, and early orchidopexy is critical for improving fertility while hormonal therapy has fewer advocates [7].

The age of orchidopexy reduced with the pathogenesis study of cryptorchidism. The recommended age reduced to below 1 year because they found germ cell loss in UDT occured at 1–2 years of age [7]. Kollin determined spermatogenesis and testicular volume by ultrasonography and demonstrated that surgery at 9 months had a beneficial effect on the growth of previously undescended testes [5, 12]. The British Association of Paediatric Urologists (BAPU) consensus meeting has set the target age for completion of orchidopexy at 6–12 months in 2011,and this recommended age is accepted by many European countries [11, 13].

Has new evidence changed practice in China? In our study, the median age from 2010 to 2015 had remained fairly static at 2 years, and the median age has not matched the target age in any subsequent year. Other studies have also shown that the average age at orchidopexy currently remains significantly older than the suggested targets and bringing the age down may be difficult to achieve [10, 13, 14].

### Why is the target not achieved and what can we do?

On an individual level, there are many reasons why there may be a delay of orchidopexy for any given patient. By examining the referral pathway for our population and the data in the survey, we attempted to determine the main factors that may contribute to a delay of orchidopexy. A large number of patients accumulated in pediatric surgery center, therefore, it was felt that the reason for delay to surgery was due to long waiting list time. In our study, the total waiting list time of orchidopexy ranged from 79 days to 1032 days. Early orchidopexy would therefore require a referral before 3 months of age.

We then considered primary care. We notice the timing of referral to a surgical center. Our GP survey showed that the delay in referral pathway was likely to be a significant contributing factor. Since most infants with UDT were diagnosed when going for baby check, it is the reassessment and timing of referral that varies greatly between GPs. Only 25.50% of them would directly make a surgical referral to pediatric surgery specifically at this point. The majority of the primary healthcare practitioners would suggest to bring children back for reassessment, usually at around 1 year, and receive the operation before 2 years old if required. It is likely that delay in the referral pathway, and limited knowledge of current targets re significant contributing factors in the failure to reach the 12-month target.

The results of our healthcare practitioners survey demonstrated that the recognition of orchidopexy age and the delay in referral pathway were likely to be significant contributing factors [10, 15]. Catherine J have demonstrated only 25% of GPs would directly make a surgical referral at their first 6-week baby check: 20% to pediatric surgery specifically and only 5% to general surgery [10]. And only 2% of the healthcare practitioners knew the recommended age of orchidopexy was 6–9 months in our survey. 14.3% of them would directly make a surgical referral to pediatric surgery specifically at this point. Thus, the healthcare practitioners should continue to have education to strengthen the update knowledge of cryptorchidism, included the age of orchidopexy and the necessity to make a surgical referral to pediatric surgery [16, 17].

Most of Chinese babies are seen by their GP after birth. This is an ideal opportunity to identify infants with UDT and refer onwards [10]. It is suggested that infants should be provided a referral to a surgical outpatient clinic at around 3 months of age. Although a small number of testes will descend between GP visit and the outpatient clinic, delay in referral after 3 months is still likely to lead to late orchidopexy.

The median age at orchidopexy throughout this time period was analyzed every 2 months, and it decreased from 24 months (1 April 2014–1 February 2015) to 12 months (1 June 2016–31 July). Tracking the age of orchidopexy revealed a statistically significant trend downward after our educational update of new transferring model. Interestingly, there was a slight decrease in percentage of orchidopexies performed before the age of 24 months in the second stage study when compared with the first stage. It was considered to owing to the interrupted educational update, and it showed a risk that the healthcare practitioners could follow again the old timing system over 12 months. Which meas, it’s important to provide continuous learning courses for referring providers and healthcare practitioners.

## Conclusion

The management of undescended testis may be improved with continuing educational updates and new transferring model among primary healthcare practitioners. To our knowledge this is the first study to report results of new transferring model and educational services for RPs designed to make age of orchidopexy earlier in China. The success of communication with RPs in achieving early orchidopexies may be applied to other types of medical management and medical education in many districts of China.
